# Association Between Keratoconus and Shoulder Dislocation: A Cross-Sectional Study

**DOI:** 10.7759/cureus.19279

**Published:** 2021-11-05

**Authors:** Walid Sharif, Khaled A Elubous, Zuhair Sharif, Saif Aldeen AlRyalat, Hashem E Al Hawamdeh, Mohammed A Abu-Rumaileh, Aws Khanfar, Muawyah D Al Bdour, Osama H Ababneh

**Affiliations:** 1 Department of Ophthalmology, Jordan University Hospital/The University of Jordan, Amman, JOR; 2 Department of Orthopaedics, Jordan University Hospital/The University of Jordan, Amman, JOR; 3 Department of Endocrinology, The University of Jordan, School of Medicine, Amman, JOR

**Keywords:** shoulder dysfunction, recurrent dislocation shoulder, joint hyperlaxity, shoulder dislocation, keratoconus

## Abstract

Introduction

Few studies have highlighted the correlation between shoulder dislocation and keratoconus (KC). This study aimed to examine the association between KC and shoulder dislocation using patients with KC and matched controls.

Methods

This cross-sectional study was conducted at Jordan University Hospital. We included patients diagnosed with KC from Jordan University Hospital's Ophthalmology Department between 2015 and 2018. We also included age- and sex-matched controls recruited randomly from fitness centers and shopping malls. All participants had complete ophthalmic and orthopedic assessments. KC was diagnosed by clinical examination followed by Pentacam (Scheimpflug Images, Oculyzer, WaveLight, Alcon, USA) confirmation.

Results

A total of 238 patients, with a mean age of 29.53 (±11.20) years, were included in this study. They were 144 (60.5%) men and 94 (39.5%) women. Moreover, 120 (50.4%) had KC while 118 (49.6%) did not have KC. Only 11 (4.6%) patients had previous shoulder dislocation. We did not find a significant difference in the frequency of shoulder dislocation between patients with and without KC (p = 0.512).

Conclusion

This study provides further evidence on the lack of association between shoulder dislocation and KC, an association that was presumed due to shared collagen characteristics.

## Introduction

Keratoconus (KC) is the most prevalent form of corneal ectasia characterized by non-inflammatory progressive central or paracentral steepening and thinning leading to myopia, irregular astigmatism, and corneal scarring [1-2]. Commonly reported histopathological findings are scarring of Bowman’s layer and anterior stroma with collagen fragmentation, fibrillation, and fibroblastic activity. Interestingly, yet underreported, studies have investigated the association between KC and joint hypermobility [3-6]. Examples of hypermobility conditions that may result in shoulder joint laxity and, thus, potentially increased risk of KC, include primary joint hypermobility syndrome [7], Ehlers-Danlos syndrome [8], and Marfan syndrome [9]. Patients with systemic hypermobility collagen disorders can also present with frequent joint dislocations and subluxations, chronic musculoskeletal pain, soft skin that may be elastic, osteoporosis, early-onset osteoarthritis, and cardiovascular abnormalities such as mitral valve prolapse [7].

Due to limited literature on this topic, this study aimed to examine the association between KC and shoulder dislocation using patients with KC and matched controls.

## Materials and methods

This study was conducted at Jordan University Hospital to assess the association between KC and shoulder dislocation. All participants provided verbal informed consent. The study adhered to the principles of the Declaration of Helsinki, and the institution’s research development committee granted ethical approval.

Patient selection

We included cases diagnosed with KC from Jordan University Hospital Ophthalmology Department between 2015 and 2018. We also included age- and sex-matched controls recruited randomly from fitness centers and shopping malls. They were screened and did not have any ophthalmic comorbidities including KC. All participants had complete ophthalmic examinations and investigations. KC was diagnosed by clinical examination, followed by Pentacam confirmation (Scheimpflug Images, Oculyzer, WaveLight, Alcon, USA).

All participants were prospectively assessed by the hospital’s orthopedic department for shoulder instability using the Beighton score to quantify joint laxity and hypermobility. Patients that scored five of nine were considered to have positive joint hyperlaxity. Patients with non-traumatic shoulder dislocations were included in the study. The exclusion criteria were other ophthalmic and orthopedic comorbidities and traumatic causes of shoulder dislocation. To further reduce bias, all participants' shoulder assessments were conducted blindly by orthopedic clinicians.

Main outcomes measures and follow-up

The main outcome measures were shoulder dislocation, spectacle dependence, contact lens use, crosslinking, penetrating keratoplasty, intrastromal corneal rings, first-degree relative, second-degree relative, third-degree relative, consanguinity, eye rubbing, dryness, smoking, vernal keratoconjunctivitis (VKC), asthma, eczema, hypertension, and diabetes mellitus. Since it is a cross-sectional study, these measures were recorded at the last follow-up visit.

Statistical analysis

We used SPSS version 21.0 (IBM Corp., Armonk, NY) in our analysis. We used mean (±standard deviation) to describe continuous variables (e.g., age). We used count (frequency) to describe other nominal variables (e.g., sex). We performed the Mann-Whitney U test to analyze the median age difference between patients with shoulder dislocation and those without and used the independent sample t-test to analyze the mean age of patients with KC and controls. We also performed the chi-square test to analyze the difference in frequency between patients with and without KC in regard to shoulder dislocation. In addition, we used the chi-square test to analyze the difference between patients with and without KC and between patients with a shoulder dislocation and those without in regard to other nominal variables. We adopted a p-value of 0.01 as a significant threshold to account for multiple variable analysis.

## Results

**Figure 1:**
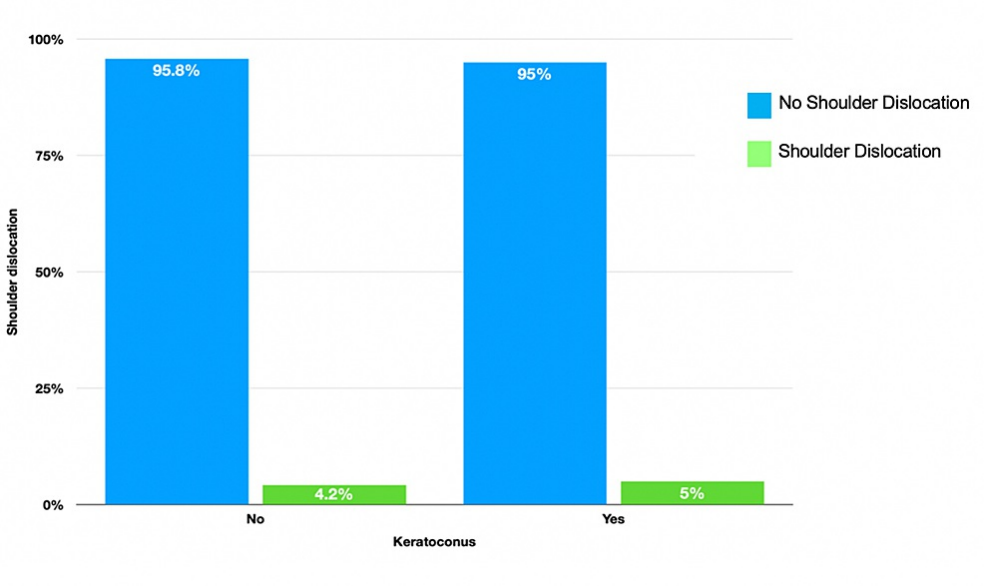
Comparison in the percentage of shoulder dislocation between patients with keratoconus and their matched controls

A total of 238 patients, with a mean age of 29.53 (±11.20) years, were included in this study. There were 144 (60.5%) men and 94 (39.5%) women. Moreover, 120 (50.4%) had KC while 118 (49.6%) did not have KC. Only 11 (4.6%) patients had previous shoulder dislocation. We did not find a significant difference in the frequency of shoulder dislocation between patients with and without KC and (p = 0.512), as shown in Figure 1. No participants had systemic collagen-related disorders.

In regard to KC, we found a significant age difference (p < 0.001), with the mean age for patients with KC as 31.60 (±13.39) years compared to 27.43 (±7.92) years for patients without KC. Table 1 presents the characteristics of patients with and without KC.

**Table 1:** Characteristics of patients with and without keratoconus Abbreviations: CL, contact lens wear; CXL, crosslinking; PKP, penetrating keratoplasty; ISCR, intrastromal corneal ring; VKC, vernal keratoconjunctivitis; HTN, hypertension; DM, diabetes mellitus

Characteristics		Keratoconus				p-value
		No		Yes		
		Count	Row N %	Count	Row N %	
Sex	Male	90	62.5%	54	37.5%	<0.001
	Female	28	29.8%	66	70.2%	
Glasses	No	88	66.2%	45	33.8%	<0.001
	Yes	30	28.6%	75	71.4%	
CL	No	117	57.4%	87	42.6%	<0.001
	Yes	1	2.9%	33	97.1%	
CXL	No	114	57.6%	84	42.4%	<0.001
	Yes	4	10.0%	36	90.0%	
PKP	No	118	61.5%	74	38.5%	<0.001
	Yes	0	0.0%	46	100.0%	
ISCR	No	118	52.4%	107	47.6%	<0.001
	Yes	0	0.0%	13	100.0%	
First-degree relative	No	114	57.6%	84	42.4%	<0.001
	Yes	4	10.0%	36	90.0%	
Second-degree relative	No	116	53.2%	102	46.8%	<0.001
	Yes	2	10.0%	18	90.0%	
Third-degree relative	No	117	52.0%	108	48.0%	0.003
	Yes	1	7.7%	12	92.3%	
Rubbing	No	115	66.9%	57	33.1%	<0.001
	Yes	3	4.5%	63	95.5%	
Dryness	No	89	54.6%	74	45.4%	0.004
	Yes	29	38.7%	46	61.3%	
Smoking	No	108	54.8%	89	45.2%	<0.001
	Yes	10	24.4%	31	75.6%	
Consanguinity	No	118	58.7%	83	41.3%	<0.001
	Yes	0	0.0%	37	100.0%	
VKC	No	118	54.1%	100	45.9%	<0.001
	Yes	0	0.0%	20	100.0%	
Asthma	No	114	51.4%	108	48.6%	<0.001
	Yes	4	25.0%	12	75.0%	
Eczema	No	114	49.1%	118	50.9%	<0.001
	Yes	4	66.7%	2	33.3%	
HTN	No	113	50.9%	109	49.1%	0.154
	Yes	5	31.3%	11	68.8%	
DM	No	114	49.8%	115	50.2%	0.584
	Yes	4	44.4%	5	55.6%	

In the analysis of the differences between patients with and without KC, we found a significant difference in regard to eye rubbing (p < 0.001), where 63 (95.5%) of patients with KC excessively rub their eyes, compared to only three (4.5%) in patients without KC. We also found that VKC did not occur in patients without KC. In addition, we found that having first-degree relatives with KC is common among patients with KC (36 [90%] compared to four patients without KC [10%]), which showed a statistically significant difference (p < 0.001). A similar correlation was noted among second and third-degree relatives. Regarding consanguinity, we found that 37 (100%) of patients with KC had a consanguinity factor compared to zero patients without KC (p < 0.001).

Asthma had a higher prevalence in patients with KC (12 [75%]) compared to those in patients without KC (4 [25%]), which showed a statistically significant difference (p < 0.001). Moreover, eczema was found in two (33.3%) patients with KC compared to four (66.7%) patients without KC, which showed a statistically significant difference (p < 0.001).

Our study also found that 75% of patients with KC had asthma (p < 0.001). There were no statistically significant differences for diabetes and hypertension among patients with and without KC (p = 0.584 and p = 0.154, respectively).

We did not find a significant difference in median age between patients with a shoulder dislocation and those without (p = 0.329). On the chi-square test, we did not find a significant sex difference between patients with shoulder dislocation (p = 0.231). Table 2 shows the characteristics of patients with and without a history of shoulder dislocation and the results of the difference analysis. In the analysis of the differences between patients with a shoulder dislocation and those without, we found a significant difference in regard to wearing glasses (p = 0.001), where 10 (90.9%) patients with shoulder dislocation are wearing glasses as compared to 95 (41.9%) patients without shoulder dislocation. We also found that having first-degree relatives with KC is significantly more common in patients with shoulder dislocation (p = 0.004), with six (54.5%) patients who had shoulder dislocation compared to 34 (15%) of patients who did not have shoulder dislocation. Moreover, eczema was found in 27.3% of patients who had shoulder dislocation compared to 1.3% of patients who did not have shoulder dislocation, showing a statistically significant difference (p = 0.001).

**Table 2:** Patient characteristics with and without a history of shoulder dislocation Abbreviations: CL, contact lens wear; CXL, crosslinking; PKP, penetrating keratoplasty; ISCR, intrastromal corneal ring; VKC, vernal keratoconjunctivitis; HTN, hypertension; DM, diabetes mellitus

Characteristics		Shoulder dislocation				P-value
		No		Yes		
		Count	Column N %	Count	Column N %	
Glasses	No	132	58.1%	1	9.1%	0.001
	Yes	95	41.9%	10	90.9%	
CL	No	196	86.3%	8	72.7%	0.196
	Yes	31	13.7%	3	27.3%	
CXL	No	192	84.6%	6	54.5%	0.022
	Yes	35	15.4%	5	45.5%	
PKP	No	183	80.6%	9	81.8%	0.640
	Yes	44	19.4%	2	18.2%	
ISCR	No	215	94.7%	10	90.9%	0.468
	Yes	12	5.3%	1	9.1%	
First-degree relative	No	193	85.0%	5	45.5%	0.004
	Yes	34	15.0%	6	54.5%	
Second-degree relative	No	210	92.5%	8	72.7%	0.054
	Yes	17	7.5%	3	27.3%	
Third-degree relative	No	215	94.7%	10	90.9%	0.468
	Yes	12	5.3%	1	9.1%	
Rubbing	No	166	73.1%	6	54.5%	0.158
	Yes	61	26.9%	5	45.5%	
Dryness	No	158	69.6%	5	45.5%	0.091
	Yes	69	30.4%	6	54.5%	
Smoking	No	191	84.1%	6	54.5%	0.025
	Yes	36	15.9%	5	45.5%	
Consanguinity	No	192	84.6%	9	81.8%	0.533
	Yes	35	15.4%	2	18.2%	
VKC	No	207	91.2%	11	100.0%	0.373
	Yes	20	8.8%	0	0.0%	
Asthma	No	214	94.3%	8	72.7%	0.030
	Yes	13	5.7%	3	27.3%	
Eczema	No	224	98.7%	8	72.7%	0.001
	Yes	3	1.3%	3	27.3%	
HTN	No	213	93.8%	9	81.8%	0.163
	Yes	14	6.2%	2	18.2%	
DM	No	219	96.5%	10	90.9%	0.352
	Yes	8	3.5%	1	9.1%	

## Discussion

KC is still regarded as a nonspecific sign representing a generalized collagen systemic disorder [6]. The oldest study in literature concluded that 50% of patients with KC had hypermobile joints [3]. Another study highlighted that 67% of patients with KC had associated connective tissue symptoms and abnormalities [4]. Morris and Woodward have concluded that subjects with KC are five folds more likely to have hypermobility of the metacarpophalangeal and wrist joints but less likely to have increased mobility of the trunk and knees [5].

However, other studies have highlighted the lack of association between KC and joint hypermobility. One study could not find a statistically significant difference in the prevalence of hypermobile joints and KC [6]. Marfan syndrome and other collagen disorders have a similar collagenous impact on ocular tissues [10], and shoulder laxity is also another common manifestation of such diagnoses. This explains why some studies found associations between this joint presentation and KC [10-11]. However, this association is not conclusive and dependent on the underlying cause for shoulder laxity. Our study did not find a statistically significant difference in the frequency of shoulder dislocation between patients with and without KC (p = 0.512).

Shoulder laxity is ultimately a nonspecific sign, where the underlying etiology can be the result of a range of other conditions or factors, entirely unconnected to a systemic collagen condition that would also present a risk factor for developing KC [12]. For instance, shoulder laxity can also be the result of previous traumas and therefore are entirely unrelated to the development of KC. Essentially, as should be foremost when examining any data regarding associations, associations do not denote causation. Instead, shoulder laxity may be related to a higher risk of developing KC but does not directly cause the ocular condition. Consequently, unless an individual also presents with other signs and symptoms of a systemic collagenous pathology that affects joint stability, KC is not directly related to shoulder laxity. Moreover, this association is only manifested when the underlying cause of the hypermobility is indeed the result of a systemic, hypermobility condition [10].

Many studies have reported different strengths of association between KC and systemic, familial, and ocular factors. Positive family history has been reported in 6-8% of cases, and its prevalence in first-degree relatives is 15-67 times higher than in the general population [13]. Our study found that first-degree relatives with KC are common among patients with KC (36 [90%]) compared to patients without KC (4 [10%]), which showed a statistically significant difference (p < 0.001). A similar correlation was noted among second and third-degree relatives. Interestingly, we found that having first-degree relatives with KC is significantly more common in patients with shoulder dislocation (p = 0.004), with six (54.5%) patients who had shoulder dislocation compared to 34 (15%) for patients who did not have shoulder dislocation.

Several studies examined the association between eye rubbing and the development of KC [14-18]. Our study has shown that 63 (95.5%) patients with KC excessively rub their eyes compared to only three (4.5%) patients without KC, which showed a statistically significant difference. This association may be due to the activation of wound healing processes and signaling pathways secondary to mechanical epithelial trauma and direct rubbing-related mechanical trauma to keratocytes and increased hydrostatic pressure in the eye [19].

We believe this study has limitations that need to be considered in future projects. Larger sample size studies are needed to further validate our findings. The retrospective nature also indicated reliance on non-study observers for KC data recording. We were unable to validate some participants’ recall of family history information as family members were unreachable. Shoulder dislocation also depends on the activities of a person; someone with a high Beighton score but a sedentary lifestyle might not have shoulder dislocation. Therefore, shoulder dislocations may have to be compared in the background of similar lifestyles in future studies.

## Conclusions

Our study highlights the lack of association between KC and shoulder laxity and thus will further add to the limited literature on this interesting topic. Unless an individual also presents with other signs and symptoms of a systemic hypermobility collagen-related disorder, KC is not directly associated with shoulder laxity.
